# Adhesion of Conventional, 3D-Printed and Milled Artificial Teeth to Resin Substrates for Complete Dentures: A Narrative Review

**DOI:** 10.3390/polym15112488

**Published:** 2023-05-28

**Authors:** Emmanouil-George Tzanakakis, Panagiotis Pandoleon, Aspasia Sarafianou, Eleana Kontonasaki

**Affiliations:** 1 Department of Prosthodontics, Faculty of Dentistry, School of Health Sciences, Aristotle University of Thessaloniki, 54124 Thessaloniki, Greece; tzanakak@dent.uoa.gr (E.-G.T.); ppandoleon@dent.auth.gr (P.P.); 2 Department of Prosthodontics, School of Dentistry, National and Kapodistrian University of Athens, 2 Thivon Str., 11527 Athens, Greece; aspasar@dent.uoa.gr

**Keywords:** 3D-prinitng, acrylic denture teeth, CAD/CAM, milling, complete dentures, adhesion

## Abstract

Background: One type of failure in complete or partial dentures is the detachment of resin teeth from denture base resin (DBR). This common complication is also observed in the new generation of digitally fabricated dentures. The purpose of this review was to provide an update on the adhesion of artificial teeth to denture resin substrates fabricated by conventional and digital methods. Methods: A search strategy was applied to retrieve relevant studies in PubMed and Scopus. Results: Chemical (monomers, ethyl acetone, conditioning liquids, adhesive agents, etc.) and mechanical (grinding, laser, sandblasting, etc.) treatments are commonly used by technicians to improve denture teeth retention with controversial benefits. Better performance in conventional dentures is realized for certain combinations of DBR materials and denture teeth after mechanical or chemical treatment. Conclusions: The incompatibility of certain materials and lack of copolymerization are the main reasons for failure. Due to the emerging field of new techniques for denture fabrication, different materials have been developed, and further research is needed to elaborate the best combination of teeth and DBRs. Lower bond strength and suboptimal failure modes have been related to 3D-printed combinations of teeth and DBRs, while milled and conventional combinations seem to be a safer choice until further improvements in printing technologies are developed.

## 1. Introduction

According to the National Health and Nutrition Examination Survey (NHANES) of the United States of America, the prevalence of edentulism (complete tooth loss) among adults aged 65 and over is 13.8% (95% CI: 10.6–17.6) [1]. Although dental implants have been proven a viable alternative to removable prosthodontics, some of the population still cannot afford the increased cost and are afraid of possible complications that surgical procedures could involve [2]. Conventional prosthodontics involving fixed partial dentures or removable prostheses are well documented and still a reliable solution for much of the population [3].

Complete dentures are usually applied as an effective and low-cost solution to satisfy the social and physiological needs of edentulous patients, by replacing the entire dentition and associated structures of the maxillae or mandible [4]. Although conventional complete dentures (CCD) are the most widely used dentures, contemporary and advanced fabrication methods have been introduced in the clinical practice to reduce denture delivery time, facilitate manufacturing process and reduce overall production costs. 

Dentures can now be made utilizing advanced technologies in addition to traditional processing methods such as heat curing and self-curing [5]. There are two fabrication processes for the construction of a digital removable denture, an additive process where the denture is fabricated by stacking the acrylic material layer by layer (3D printing) or a subtractive one, such as computer-aided design and computer-aided manufacturing (CAD/CAM), where the material is milled with specific cutting tools that are guided through computer numerical control technology [5,6]. Three-dimensional printing can be achieved by either stereolithography (SLA) or digital light processing (DLP), and both processes achieve a satisfying precision of 3D-printed resin layers (20 to 150 µm) [7]. In both processes, the acrylic denture base is initially milled or printed first, and the traditional CAD/CAM or 3D-printed denture teeth are attached using a bonding agent [8,9]. 

There are several methods for attaching denture teeth to a denture base. In CCD, heat polymerization, self-polymerization and microwave heating have been proposed, although heat polymerization has been associated with stronger bonds [10]. The attachment of the denture teeth to the denture base resin (DBR) occurs during the heat polymerization stage, while in digital workflows, teeth can be either printed or milled separately from the denture teeth and subsequently bonded to the printed acrylic base. Additionally, for better aesthetics, certain processes permit gluing acrylic resin denture teeth to a printed denture base. According to Schneider et al. [10], choosing more suitable combinations of acrylic teeth and DBRs may lower the incidence of prosthesis failures and the ensuing need for costly and time-consuming patient repairs.

A significant issue with complete dentures is the debonding of teeth from the denture base resin. According to estimates, tooth debonding occurs in 22–30% of conventional denture restorations, particularly at the area of the anterior teeth [11]. The physical contact of the denture tooth with the polymerizing denture base resin and the reaction between the denture base resin’s polymer network and the acrylic tooth material to form an interwoven polymeric network determines the bonding effectiveness between the acrylic teeth and the denture base resin. Appropriate expansion of the adhesive area through surface treatment to the denture tooth by micro-retentive (e.g., alumina sandblasting) or macro-retentive patterns (grooves, inverted cone) increases the contact area between the teeth and denture base, providing mechanical interlocking [12]. 

In a purely theoretical approach, the ideal bond could only be made under the condition that base and tooth material were simultaneously polymerized during the manufacturing process of the prosthesis in one step. Although some attempts were made in the past, it was not possible to achieve satisfactory aesthetics in the acrylic teeth [13]. Therefore, there remains the difficulty of connecting a pre-polymerized acrylic tooth with a material that must polymerize satisfactorily but at the same time have the possibility to undergo copolymerization (either through diffusion or through free -C-C- bonds) and micromechanical retention with part of the acrylic tooth. Based on this philosophy, most researchers’ efforts to improve the bond have been developed with mechanical or chemical modifications to part of the artificial teeth and will be discussed later on [14].

Since the application of osseointegrated implants has expanded, the role of acrylic resin in implant constructions is also important. Jemt reports that, in patients with prosthetic restorations on implants, fractures of acrylic teeth are the most common complication [15]. It should also be noted that with the introduction of osseointegrated implants into daily clinical practice, the types of prosthetic restorations multiplied, and the further use of acrylic resin also expanded in implant restorations. From these observations, we conclude that the importance of the bond between the acrylic resin and the acrylic base is of value in constructions such as overdenture implants [16] and hybrid constructions on implants, which, due to the increased chewing loads they receive, must have a high strength in order to be a reliable solution for clinicians. 

Commercially accessible DBRs and teeth come in a wide variety for denture manufacture. However, the process used to make the denture teeth may have an impact on how well they adhere to the DBR. Based on the manufacturing process, the varying bonding strengths or compatibility of acrylic teeth with DBRs can be assessed by ISO standards, which describe the requirements for materials and their testing methodology (ISO 20795-1:2013(en) Dentistry—Base polymers—Part 1: Denture base polymers or ISO 22112:2017(en) Dentistry—Artificial teeth for dental prostheses, ISO/TS 19736:2017 Dentistry—Bonding test between polymer teeth and denture base materials). Due to the novelty of CAD/CAM (described as a subtractive technology) and 3D printing (an additive technology manufacturing process) for denture manufacture, investigations to confirm the effectiveness of 3D-printed and CAD/CAM-milled denture resins in bonding to artificial denture teeth are not yet available. Whatever the method, it is crucial to take the bond strength into consideration.

The purpose of this review was to provide an update on the adhesion of artificial teeth to resin substrates from the most recent methods for conventional and digital fabrication of complete dentures and discuss factors affecting bond strength in conventional and contemporary fabrication methods. All the abbreviations reported in the text are listed in Appendix A.

## 2. Materials and Methods

Although this was not a systematic review, a systematic method was applied to retrieve the most relevant literature. We searched PubMed and Scopus without a time frame. The search terms used were: (complete denture teeth) and (bond strength), (complete denture teeth) and (adhesion), (3D printed teeth) and (complete denture), (CAD-CAM teeth) and (complete denture), (milled teeth) and (complete denture), (acrylic teeth) and (complete denture), (denture teeth) and (denture base) and (adhesion), (denture teeth) and (denture base) and (bond strength). Inclusion criteria were applied to retrieve only in vitro studies written in the English language. Exclusion criteria were applied to exclude studies not reporting data regarding adhesion or bonding and debonding of complete denture teeth and materials, and studies evaluating removable partial dentures or partial dental prostheses. Hand searching was also performed on all the included articles and relative reviews.

## 3. Results and Discussion

### 3.1. Adhesion of Conventional Teeth to Conventional Denture Base Resin

**Figure 1:**
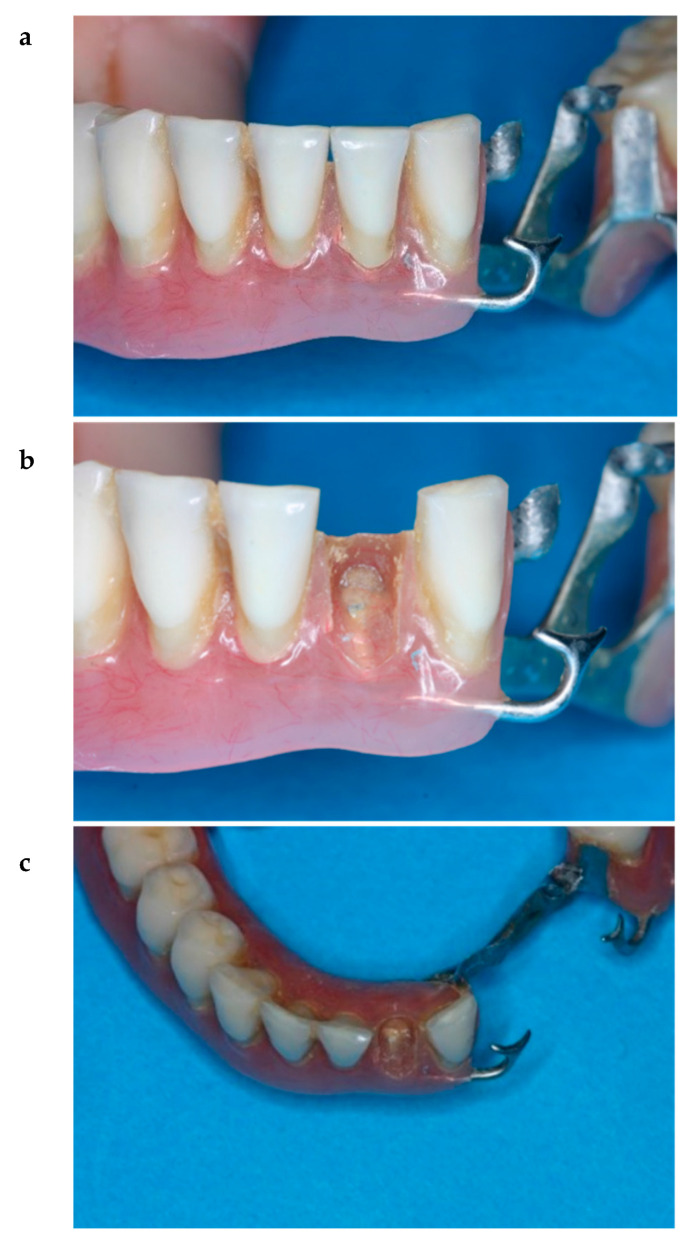
Images of a detached tooth from the acrylic resin of a removable partial denture after one year of use ((**a**). frontal view, (**b**). frontal view of detached acrylic tooth #32, (**c**). occlusal view of detached tooth #32). The repair could involve an inverted cone inside the acrylic tooth, which many dental technicians use before pouring heat-cured acrylic.

From a clinical perspective, tooth detachment from a complete or removable partial denture, especially in the anterior area, results in discomfort to the patients and is a main reason for urgent visits to the dentist (Figure 1). The reasons for tooth detachment might be manufacturing errors during the fabrication process, issues related to materials (teeth and DBR) or mastication overload [17]. Greater patient satisfaction can be derived from reliability in long-term prostheses [18].

It was stated that an extremely high number of repairs occurs, exceeding 60% of the number of dentures manufactured each year [19]. A similar finding is also seen in other studies [11,20] where it was concluded that a very common type of repair concerns the detachment of denture teeth, both in complete and partial dentures. Although excessive occlusal load on denture teeth during mastication can be responsible for teeth detachment, other reasons have been reported in the literature. One of the most likely explanations for the failure of the bond between the acrylic teeth and the denture base was believed to be the contamination of the tooth surfaces [21], which prevents a strong bond from being achieved. Contaminants are basically traces of wax absorbed from the tooth surfaces during acrylic processing, or spacer material left at the base of the teeth in the acrylic packing stage. Contrary to many researchers [21,22,23], Spartley found that the presence of spacer material on the teeth did not affect bond strength under the condition that wax elimination was conducted at least at 90 °C [24]. Debonding could be caused by mismatched surface conditions at the tooth and base interface. This incompatibility is thought to be caused by two things: (a) contamination of the involved surfaces or (b) structural differences between the two components due to processing methods [19,20,21,22,23,24,25]. It has been estimated [11,26] that 22–30% of denture repairs concern tooth debonding, typically in the anterior portion of the denture. This is despite the statistics not distinguishing between fracture and debonding.

Strongly cross-linked teeth have reduced bond strength, and employing high-impact resins significantly improves tooth bonding. Unfortunately, there is no agreement on how altering the tooth ridge lap or the alginate mold seal on a tooth can affect the bonding of denture teeth. Bonding has often been found to suffer from residual wax on the tooth surface [19]. In clinical practice, Kurt et al. [27] proposed that bonding strength was modified by wax removal techniques. The best outcomes were obtained when MMA monomer and wax solvent were applied to the ridge lap surfaces of the teeth. In addition, the application of MMA and wax solvent independently increased the bonding between acrylic denture teeth and base resin. The strongest bond was produced when wax solvent was used before applying monomer. Although the application of MMA and wax solvent independently increased the bonding between acrylic denture teeth and base resin, the strongest bond was produced when wax solvent was used before applying monomer. 

The fact that copolymerization was not a critical factor for reducing bond strength was noticed in 1958 [28]. Denture base acrylic resin, during its polymerization, comes into physical contact with the artificial teeth, and the polymer network of the base chemically reacts to form an interwoven polymer network. The compatibility of the two polymers affects the success of the chemical bond and, consequently, the strength of the bond [10,29]. Another reason might be the regular use of disinfectant or cleaning chemical substances, which can change the physio-mechanical properties of both DBR and teeth [30,31], leading to weakening their bond and subsequent detachment [32].

Materials-related factors affecting the bond between artificial denture teeth and DBR materials include differences on the denture base composition and polymerization type, the type of acrylic teeth and the type of their interfacial surface pretreatment. The most well-known mechanism controlling the bonding process is the swelling of the polymer caused by the diffusion of an appropriate solvent. Diffusion depends on time, temperature, type of solvent and the structure and glass transition point of the polymer [33]. Because denture teeth are pre-polymerized, the chemical co-polymerization that could create an interconnected polymer network with any denture base material is difficult, since free-radical concentration is very low [34]. Moreover, the adhesion of denture base resin to denture teeth has been explained through the involvement of unreacted methyl-methacrylate groups, which in cold-cured materials cannot be proven because the remaining double bonds do not react at room temperature [35]. Although heat-cured acrylics reach a high degree of double-bond conversion and, therefore, more reactive free radicals are created, the low free radical concentration in pre-polymerized acrylic teeth does not ensure chemical bonding to acrylic denture bases [26]. To compensate for this problem, various strategies have been attempted to increase the interfacial adhesive bond strength by creating either micro- or macromechanical interlocking or some kind of chemical reaction between the different types of polymers. 

#### 3.1.1. Methods to Increase Denture Base to Acrylic Teeth Bond

Researchers have employed many different approaches to enhance denture base–acrylic teeth adhesion. Most of them require surface pretreatment in the adhesive area of the denture tooth. The type of artificial teeth and the material of the denture bases affect the strength of the bond [36,37,38]. In other words, the chemical composition of the pre-polymerized artificial denture tooth influences the surface treatment protocol. Moreover, denture base material composition and polymerization methods also influence adhesive potential to prefabricated artificial teeth. In general, the strategies to alter the surface of an artificial prefabricated tooth are based on the theory of micromechanical retention, chemical co-polymerization and the management of the polymerization shrinkage of polymers.

##### Chemical Treatments

Chemical treatments are based on the theory that the polymerizable monomer plasticizes the surface of the acrylic tooth and diffuses into the acrylic resin of the tooth. Monomers derived from the polymerized acrylic resin of the base material penetrate the acrylic resin of the artificial teeth and “swell” its surface [33]. The thickness of this layer, which comes from the reaction of the monomers with the polymer grains and the PMMA matrix, seems to be related to the strength of the bond between the acrylic resin of the teeth and the acrylic resin of the base. During the polymerization of the monomer, a network of polymer chains is formed that connects the two polymers [5]. 

Different chemical modifications have been attempted to increase adhesion between teeth and denture base materials (Table 1).

Many researchers have tried to treat acrylic teeth with liquid methyl methacrylate (MMA) [23,24,27,29,84] or a combination of MMA with methylene chloride [19,29]. Spratley et al. [24] concluded that placing monomer on the cervical portion of the teeth did not appear to affect bond strength. Similarly, in the study by Barpal et al., surface treatment of acrylic teeth with MMA monomer either reduced or had no effect on the bond strength of the thermosetting acrylic resin to the denture base when placed for thirty seconds before layering the resin [84]. Morrow et al. showed that placing a monomer–polymer solution on the unmodified cervical part of artificial teeth reduces the bond strength [23], in accordance with Rupp et al., who reported that placing monomer on the tooth surface reduces the bond strength of a self-curing acrylic resin [29]. Palitsch et al. [71] found that the application of MMA in combination with the investigated light-cured DBR materials resulted in insufficient bond strength, in accordance with the literature [36,91,92]. They attributed their findings to the restricted penetration of DBR in the MMA dissolved tooth surface, the high viscosity of DBR, the wetting issues that blocked sufficient micromechanical locking or the inadequate copolymerization of MMA and the bifunctional monomers of the light-cured DBR materials. These differences in results are due to the different experimental methods and denture base and teeth materials. 

Many different chemical agents have been used, apart from MMA. Sorensen and Fjeldstad [93] reported that bond strength is improved when artificial acrylic teeth are treated with appropriate solutions (ethyl acetone or monomer). Takahashi et al. [5] reported on the effect of dichloromethane (CH_2_CL_2_—an organic solvent that dissolves the polymer matrix of PMMA) in improving the retention of both conventional teeth and cross-linked acrylic teeth. In a similar study, it was demonstrated that dichloromethane improved the tooth bond strength of both conventional and highly cross-linked acrylic teeth to three times the initial value of untreated teeth [94]. Dichloromethane causes an increase in the external surface volume of the acrylic resin of the teeth, allowing the polymerizable acrylic resin monomers of the base to permeate the artificial teeth and form an extensive interwoven polymer network. The microroughness exhibited by dichloromethane-treated tooth surfaces that leads to increased mechanical retention may be involved in the higher bond strength [33,65,93,95]. 

Suzuki et al. [96] noticed that the bonding was greatly enhanced by applying 4-META adhesive agents to highly cross-linked teeth prior to packing the resin dough. Fletcher-Stark et al. [73] reported that the use of a bonding agent (Eclipse; Dentsply), lead to significantly increased bond strength in IPN denture teeth, only when it was applied in combination with a light-polymerized UDMA resin, and not in combination with heat-polymerized PMMA resin. Palitsch et al. [71] used different conditioning liquids combined with different denture base materials and evaluated the tensile bond strength to denture teeth composed of PMMA (80%) and additives (ethylene glycol-dimethacrylate and pigments). They used an acetone-rich agent, which did not present satisfactory results, probably due to the early evaporation of acetone. They concluded that light-cured materials need special bonding liquids, consisting of appropriate mixtures of solvents, to adequately treat the teeth surface for effective bonding.

Another bonding agent applied for bonding DBR materials to acrylic teeth is methylacetate, under the commercial name Eclipse Bonding Agent (Eclipse; Dentsply). Its use in the study of Akin et al. [97] did not result in optimum bond strength when combined with a heat-polymerized DBR, while in another study by the same authors [67], its use led to significant improvement in bond strength when combined with light-cured DBR materials. The same bonding agent was used and compared to a diclhoromethane-based bonding agent to test its bonding efficacy to a light-cured DBR, and it proved to be effective only when combined with a mechanically roughened surface [98]. Nishigawa et al. [82] used a bonding agent consisting of MMA at 85%wt. and a low-molecular-weight polyethylmethacrylate (PEMA) at 15% wt. They concluded that the applied bonding agent not only was effective to increase the shear bond strength of DBR with artificial denture teeth, sandblasted or not, but the bond was the highest even after 100 days in water; the sandblasted interfaces with additional bonding agent particularly retained their strength after water storage. Perea et al. [99] tested four different monomer systems to evaluate the shear bond strength between PMMA DBR and acrylic resin denture teeth: flowable composite resin, MMA, composite primer and stick resin. Except for MMA, all materials contained a photopolymerizable initiator system. The authors concluded that as long as the monomer systems are allowed to adequately dissolve the polymer network of acrylic teeth, a strong bond can be achieved. 

##### Mechanical Treatments

Moreover, macromechanical retention and roughness adjustments have been proposed to enhance the total contact surface area between the two polymers (Table 1). Sandblasting is a traditional method to increase the surface contact area and is very common in base metal and zirconia restorations before cementing [100]. It seems that this method increases bond strength to both acrylic and ceramic denture teeth [64,101]. Other concepts include the modification of the adhesive area by grinding, or by creating mechanical macro-retentive patterns [83,95]. It was found that vertical grooves minimize stress concentration at the tooth–base interface [11,86]. It seems that two parallel grooves and one retention hole increase the tensile strength of acrylic teeth to the denture base material [26,84,95,102,103,104]. In the study by Akin et al. [97], researchers did not make grooves on the bonding surface but instead ground it with a tungsten carbide bur to create areas with macro-retention. They also evaluated Er:YAG laser and airborne-particle abrasion with 120 μm Al_2_O_3_ particles at 2 bar pressure for 10 s as alternative mechanical treatments. They concluded that all types of mechanical pretreatment constitute effective ways to significantly improve bond strength. Chung et al. [79] combined sandblasting with 250 μm Al_2_O_3_ particles and grinding, and reported that this combination can provide significantly higher bond strength due to the increased surface area and mechanical interlocking. On the contrary, Cardash et al. [95] found both an increase and a decrease in bond strength in a mechanically modified cervical portion of artificial teeth. Cunningham et al. also reported that the formation of grooves and grinding of the tooth surface can be effective in the absence of thorough dewaxing [83]. 

#### 3.1.2. Effect of Materials on Bond Strength

##### Denture Base Materials

Denture base materials are basically classified by their type of polymerization [105]. Heat-cured, cold-cured, visible light-cured, microwave-cured and pour-type denture base materials have been developed. In terms of materials selection for their fabrication, the most common material for DBR is poly(methyl methacrylate) (PMMA), although it has low mechanical strength. To further improve the mechanical properties of PMMA DBR materials and prevent fractures, different additives have been tested, such as glass, rubber, polyethene and polypropylene fibers, and alumina, zirconia, titanium, silver, silica-based and hydroxyapatite fillers [106]. Another drawback of PMMA is that it has an allergenic potential in patients with sensitivity to methyl methacrylate monomer [107]. As an alternative to PMMA, light-polymerized resins were developed, based on di-methacrylates such as Bis-GMA, UDMA and TEGDMA [108], which, however, presented lower bond strength in denture teeth [87,88,109], leading to the necessity of applying bonding agents. Composite materials, however, provide easier processing and lower hyperallergic reactions [107,108]. Depending on the denture base material, particular conditioning liquids are recommended for the chemical pretreatment of resin denture teeth. The most crucial mechanism of action of a conditioning liquid is thought to be that it penetrates the bonding surface, solubilizes and/or swells it and ultimately results in a chemical link to the denture base material [79,83,110].

Denture teeth generally bond better with heat-cured acrylic resins than with self-curing acrylic resins. It has been mentioned that the polymerization of heat-cured acrylic resins is more complete than that of self-curing acrylic resins [89], while others believe that the higher polymerization temperature in heat-cured acrylic resins increases the diffusion of monomers to the teeth and thus bond strength is improved [10,33,111]. In another study and according to Cardash et al. [95], high-impact strength heat-cured acrylic resins showed better retention than conventional ones, but the research by Morrow et al. failed to show a statistically significant difference between them [23]. According to certain studies, the bond strength of artificial teeth to heat-cured resins is stronger than that to the self-cured type [84,88,89]. Takahashi et al. [5] used heat- and microwave-processed DBR and compared them with a poured PMMA DBR resin. The best results were observed with the heat-cured DBR, with significant difference, while both heat-cured and microwave-processed DBRs showed higher bond strength compared with the pour-type resin. Similar were the findings from tensile tests performed by Damade et al. [112], who evaluated the adhesion of cross-linked acrylic teeth by heat and microwave polymerization. Palitsch et al. [71], using various conditioning solutions, compared the bond strength among conventional MMA/PMMA and two light-curing denture base materials with acrylic teeth. The findings showed that the best results in terms of bond strength to resin-based denture teeth come from using conventional MMA/PMMA denture base resins. Using MMA to condition the teeth provides the most credible outcomes. Since it inhibits bonding, MMA should not be used as a conditioning liquid for light-curing denture base materials. To provide a sufficient connection to resin denture teeth, a certain conditioning liquid is required for light-curing resin base materials.

##### Denture Teeth Material

The materials employed for artificial teeth in removable complete or partial dentures are mainly heat-activated poly(methyl methacrylate) (PMMA) grains located in a matrix of cross-linked PMMA [113,114], highly cross-linked polyacrylic resins with homogeneously distributed inorganic microfillers polymerized into the matrix (MRP) [115], interpenetrating polymer networks (IPN) [116] or nanohybrid composite resins consisting of a combination of UDMA matrix with inorganic SiO_2_ fillers and PMMA clusters [117,118]. The most recent class of denture teeth are the nano-hybrid composite teeth (NHC), which contain UDMA matrix, inorganic fillers (highly dispersed SiO_2_), matrix-based pre-polymer and PMMA clusters [119]. These materials must meet certain specifications to be used in clinical practice. Artificial teeth must present good aesthetics and natural appearance and resemble natural teeth in shape, color and transparency [120,121]. 

The different types of artificial teeth and the different materials of denture bases affect the strength of their bond. In recent years, highly cross-linked artificial teeth have been introduced, which show significantly improved properties such as fracture resistance, abrasion resistance and color stability. However, conventional acrylic teeth achieve a better bond to the resin base of the denture than highly cross-linked acrylic teeth. To promote copolymerization manufacturers during the production of acrylic teeth, the cervical part contains a less cross-linked polymer. According to Clancy and Boyer [89], in conventional acrylic teeth, there are more non-crosslinked polymer chains available for bonding to the denture base. Clancy et al. [87] evaluated the bond strength of a light-cured and a heat-cured resin combined with conventional acrylic teeth and those that were abrasion resistant. The best combination was the heat-cured resin and acrylic teeth, presenting higher bond strength compared with the IPN abrasion-resistant teeth. The bond strength with the light-cured resin and both tooth combinations was consistently low. In the study of Chai et al. [94], a pour-type DBR was bonded to conventional and cross-linked denture teeth and was evaluated before and after thermal cycling. The authors proved evidence that there were no significant differences between the groups, nor before or after thermal cycling, while they emphasized the efficacy of dichloromethane application in improving the bond strength even after thermal cycling. Dichloromethane solvent results in the swelling of denture teeth resin, so monomers can penetrate deeper, strengthening their network. It also leads to surface micro-roughening, which further enhances micromechanical bonding. 

It would be a significant project to evaluate the bonding strength of each combination given the wide variety of tooth and denture base products available on the market. Assessing the bonding qualities of “matched” and “mismatched” combinations of teeth and denture base material may be a more successful strategy. In the literature, there is limited evidence that higher bond strength can be achieved when both DBR and denture teeth are made by the same manufacturer [122,123,124]. In the study of Colebeck et al. [124], the authors evaluated NHC teeth with different DBR materials and concluded that these teeth produce significantly lower bond strength when combined with denture bases of other manufacturers, despite their chemical similarity. 

In summary, there is a controversy on what combination works better, and different studies show completely opposite results. Although it has been proven that the bond strength of composite teeth to the acrylic base material is higher than that of acrylic teeth [66], other studies reported that the bond strength of acrylic teeth is higher than that of composite teeth [125,126]. 

### 3.2. Adhesion of CAD/CAM-Milled and 3D-Printed Teeth to Denture Resin Base

**Figure 2:**
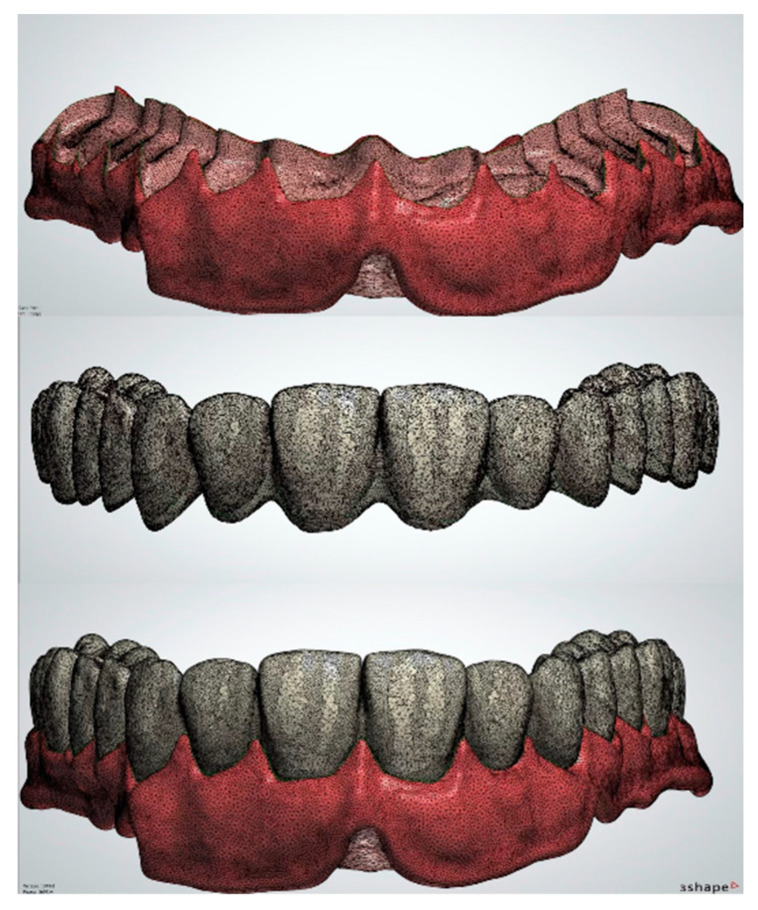
Digital design of denture base (**top**), teeth (**middle**) and final denture (**bottom**).

During conventional denture fabrication, the bonding between denture bases and teeth occurs through PMMA polymerization in contact with the artificial teeth and the creation of an interwoven polymer network. Digital denture workflow, however, usually involves the digital design (Figure 2) and separate fabrication of bases and artificial teeth, followed by bonding with the use of a bonding agent, surface conditioning or the simple application of autopolymerizing PMMA resin [45]. 

**Figure 3:**
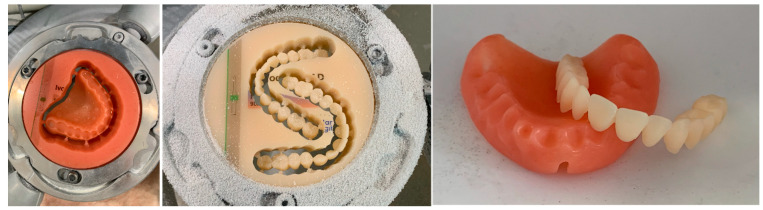
CAD/CAM denture base (**left**) and teeth (**middle**), still attached to their respective PMMA disks in the milling machine and milled denture base and teeth before bonding (**right**).

Prefabricated or CAD/CAM-milled teeth are either attached to the denture base with an adhesive or bonded using cold- or heat-polymerized PMMA [46,127] (Figure 3). 

**Figure 4:**
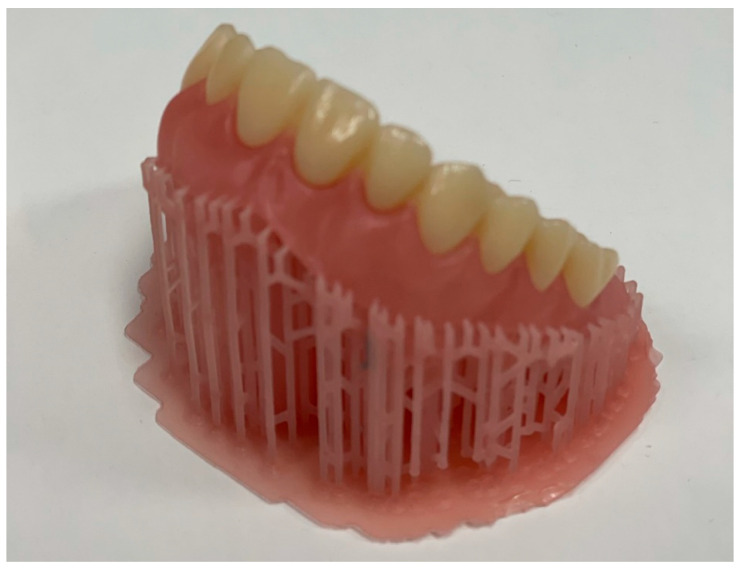
Three-dimensional-printed denture base and separately printed denture teeth connected to the denture base.

Regarding 3D-printed (additively manufactured) dentures, the denture base is printed, followed by printing the teeth in a separate process (Figure 4). The denture teeth can be connected to the denture base using a special bonding agent or unpolymerized base resin, which is subsequently light polymerized. Teeth may be either individually bonded or fused together and bonded as one piece. Artificial denture teeth may also be bonded to a printed denture base using special bonding agents and sealers [128]. 

**Figure 5:**
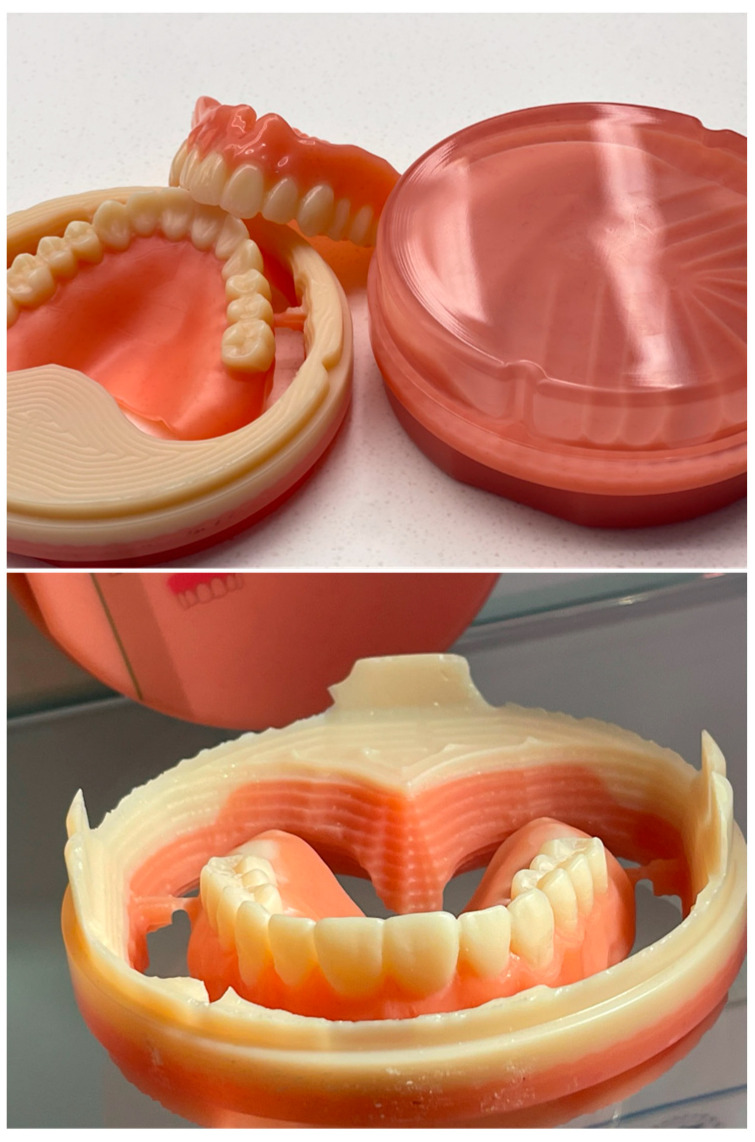
Monolithic digital denture milled out of single pink-and-white-colored disk in a single milling procedure.

Monolithic digital denture solutions have recently been developed. Manufacturers propose tooth and denture base materials (both high-grade PMMA) in two-colored discs (pink and white), for the base and teeth to be milled in a single milling process (Figure 5). Both materials are polymerized together during the industrial fabrication process, forming a direct chemical bond and eliminating the need for additional bonding materials and procedures.

A few studies have been published reporting on the bond strength of 3D printed or milled teeth with denture resin (Table 2). 

The bond between printed denture bases and separately printed teeth is inferior to that developed in conventional processing, according to the recent literature [44,47]. According to one study, the printed group experienced both cohesive and adhesive failures, while the traditionally processed group only experienced cohesive failures, suggesting that the traditionally processed denture group had a stronger connection [44]. Printed dentures appear to have a lower bond strength, despite the lack of research on the subject. To evaluate whether there is any potential clinical importance, further studies are required. A comparison of the various bonding methods should be performed, as several bonding protocols have been proposed.

Currently, there is a small number of in vitro studies comparing the bond strength of denture teeth between conventional heat-processed, CAD/CAM-milled and 3D-printed denture bases. Choi et al. [47] compared four distinct types of commercial denture teeth (PMMA, cross-linked PMMA, PMMA with nanofillers and 3D-printed) and three different types of denture base resins (DBRs) (heat-cured, CAD-milled and 3D-printed). They concluded that the highest bond strength and fracture toughness is still produced by heat-polymerized denture base resins, despite the growing popularity of CAD-milled and 3D-printed materials.

Alharbi et al. [44] evaluated the failure load between heat-cured PMMA and commercially available denture teeth and 3D-printed denture resin material before and after dynamic loading. The fabrication technique affected the mode of failure; the conventional group had mostly cohesive failures, which may indicate a stronger bond between the teeth and denture resin in this group. The recorded failure modes show that the bond strength of both procedures was adequate, while the strength of the bond was not significantly impacted by aging in the chewing simulator. The mixed mode of fracture in the 3D-printed group was attributed to either debonded areas or porosities among the printed layers or inconsistency with flaws and voids within the bonding layer. The authors also reported that the direction of loading (parallel or with inclination to the printed layers) may result in different types of failure and deformation of the materials. 

In another study, Kane et al. [45] concluded that the long-established heat-processed denture base resins are similar in performance to milled ones, which, in turn, are superior to 3D-printed ones, as evaluated using the shear bond stress test. While the shear stress for all groups was higher than the published maximum biting force of complete denture patients, all resin materials employed in this investigation may be clinically appropriate for complete denture users. Gad et al. [129] reported that, in comparison to heat-polymerized resin, 3D-printed resin showed lower values of flexural and impact strength, as well as hardness. Helal et al. [41] investigated the surface roughness and impact strength of either milled or 3D-printed materials for DBRs and explained the lower surface roughness and highest hardness of milled materials by their better homogeneity due to the high fabrication temperature and pressure, which yield a higher degree of conversion-reducing residual monomer, less porosity and less shrinkage [130,131]. 

Han et al. [132] evaluated the shear bond strength between resin denture teeth with various compositions and denture base resinous materials, including conventional and CAD/CAM materials. The results revealed that the shear bond strength values of the conventional group, with heat-polymerized PMMA denture resin, and the CAD/CAM denture base group were similar. In the study, it was also pointed out that composite teeth result in inferior shear bond strength in all tested types of DBR. According to ISO 19736, for an acceptable bond, the mode of failure should be <33% of the adhesive type, and ideally, all types should be of cohesive failure. Mohamed et al. also verified that despite differences in bond strength, all tested combinations—3D printed, milled or conventional—provided sufficient bonding capability [43].

The shear bond strengths of different denture base resins to different types of prefabricated teeth (acrylic, nanohybrid composite and cross-linked) and denture teeth produced by CAD/CAM were compared by Prpić et al. [46]. The findings revealed that the cold-polymerized resin had the lowest values across the various polymerization techniques. The shear bond strength values of CAD/CAM (milled) denture base resins and heat-polymerized resins did not significantly differ from one another. Similar shear bond strength values were seen when milled and heat-polymerized denture base resins were bonded to several kinds of prefabricated teeth, suggesting that the important factor affecting bond strength was the polymerization process of the DBR. As the bonding strength of removable denture bases and denture teeth made from various material combinations might differ remarkably, the authors concluded that when connecting prefabricated teeth to a denture base, cold-polymerized resin should be avoided.

The effect of the different bonding agents on the bond strength of denture teeth was evaluated by Cleto et al. [42]. The results of this study showed that the bond strength was affected by the type of artificial teeth and the bonding agent. The following two artificial teeth models were assessed with 3D-printed DBRs, prefabricated PMMA and teeth also produced by the printer. The PMMA teeth in the control group were attached to a typical heat-polymerized denture base resin as in conventional approaches. The results revealed that only with the MMA monomer combined with the Cosmos TEMP bonding resin, the bond strength of the PMMA teeth was much higher and comparable to the control. This combination also presented significantly higher bond strength when compared with the bonding agent Cosmos TEMP alone.

In addition, the impact of different surface treatments on the shear bond strength (SBS) was evaluated by Helal et al. [41]. They studied two different forms of DBRs (milled and heat-polymerized), as well as two different kinds of denture teeth (PMMA and composite). Four subgroups of teeth were created based on how their surfaces were treated: dichloromethane (DCM), air abrasion (AB), bur roughening and no treatment (control). The findings indicated that compared to other groups, dichloromethane application and the blasting of alumina improved the SBS of acrylic teeth to heat-polymerized DBR. In contrast to SBS with composite teeth, all surface treatments for CAD/CAM DBR with acrylic teeth revealed no measurable difference. When compared to composite teeth, acrylic teeth demonstrated an increase in SBS that was statistically significant. Although the application of alumina sandblasting and the use of DCM improved the SBS for the combination of acrylic teeth–heat-polymerized DBR, his was not the case with the CAD/CAM DBR, where the different treatments failed to reveal a significant impact. Moreover, none of the employed treatments could increase the SBS when composite teeth were combined with CAD/CAM DBR. The only treatment that positively affected the SBS of composite teeth was alumina blasting, when combined with heat-polymerized DBR.

## 4. Discussion

This review summarized the findings of in vitro studies where the bond strength between conventional and contemporary combinations of artificial denture teeth and denture base materials was evaluated. The literature is abundant when it comes to conventional materials and methods, while it is still limited regarding CAD/CAM and 3D-printed teeth. However, there are important differences in terms of experimental design, specimen fabrication, materials combinations involved, samples size, etc., that make the results difficult to homogenize and generalize.

For the conventional fabrication of complete dentures, denture base resins from methyl methacrylate monomers can be polymerized to PMMA via a number of methods, such as with heat, chemicals, visible light and microwave energy. Despite being fast, the polymerization is never complete, and in the end, a little amount of free monomer can still be seen. Additionally, porosities may be present in heat-polymerized dentures as a result of poor mixing, excessive heating, evaporation of unreacted monomer or insufficient pressure during polymerization. On the contrary, milled dentures are fabricated from pre-polymerized PMMA pucks with high density and minimal shrinkage and porosity, while they are free of unreacted monomer due to a higher degree of conversion and better polymerization. It has been reported that bond strength between teeth and DBRs may be affected by the amount of free monomers during processing, and by that, it can be concluded that conventional processing may be beneficiary to achieve a stronger bond compared with the bond achieved with pre-polymerized CAD/CAM materials [18,110]. Choi et al. [47] reported that, following the manufacturers’ instructions for the fabrication of conventional and CAD/CAM complete dentures, there is a 2.5 times larger concentration of free monomers flowing in the interface initiating the bonding process in the case of heat-polymerized dentures compared to pre-polymerized CAD/CAM, explaining the worst results in terms of bond strength in their study for their CAD/CAM specimens.

The composition of 3D-printing resins is completely different, as most of the 3D-printed resins contain photopolymerizable acrylate monomers with incorporated oxide-based photoinitiators for the efficient curing and color stability of the material, stabilizers and pigments. Indicative resins and their compositions are presented in Table 3.

By the manufacturing process that builds the denture through the layer-by-layer addition of the material, flaws and pores can be induced from the inadequate packing of the layers, which may influence the mechanical performance of the bonded interface. It has been suggested that the orientation of applied loads in relation to the printing configuration may enhance the debonding of artificial teeth, and usually, the type of fracture is adhesive at the interface [47]. Cleto et al. [42] suggested that the use of a methyl methacrylate monomer can be more effective compared with other bonding agents, such as autopolymerizing or 3D-printing resin. As there are no guidelines on how to bond 3D-printed teeth to 3D-printed DBRs yet, further research is needed to identify optimum protocols. Furthermore, the effect of DBRs’ chemical composition on bond strength should be investigated in well-designed in vitro studies to identify possible composition-related factors that could help manufacturers ameliorate the bonding performance of 3D-printed dentures.

Many international standards describe methods for determining the bond strength between acrylic teeth and denture bases. The lack of a universally accepted standardization means that each specification has some drawbacks, and each fails to be universally accepted. Combined shear, compressive and tensile forces are applied on denture teeth during mastication, and for this reason, various testing methodologies including shear [66,133], tensile [77] or micro-tensile [124], flexural and compressive [5] strength tests, as well as the chevron notch beam method [47], have been employed in evaluating the bond of artificial teeth to DBR. Additionally, different inclinations of teeth in terms of applying loading, types of teeth, geometry and cross-sectional bonding areas can affect the uniformity of applied stresses [47]. In general, simplified shear test methods have been applied [17] and only a few tensile tests have been performed. Shear test have been criticized as not appropriate to evaluate adhesive bond strength, but only the cohesive strength of the base material is configuration dependent [134], while tensile tests predict the clinical performance of the materials more accurately, as adhesive debonding is performed by tensile rather than shear forces [135]. Damade et al. [112] performed both shear and tensile tests to evaluate the bond strength between cross-linked denture teeth and heat-cured resin either with microwave or heat polymerization. They found different results in terms of bond strength based on polymerization, and statistically significant differences were produced only from the tensile test. Another parameter that adds to the difficulty of comparing the results among studies is the preservation of samples before testing. Other studies included immersion in water for different time points, other thermal cycling with different immersion time, dwell time and number of cycles.

From the survey in the relevant literature, better performance in conventional dentures is realized for certain combinations of denture base materials and denture teeth with mechanically or chemically treated surfaces. It seems that heat-cured DBRs perform better than self-cured DBRs when combined with conventional or highly cross-linked acrylic denture teeth. With regard to mechanical treatments in acrylic denture teeth surfaces, sandblasting and macro-retentive patterns are effective in enhancing the tooth–denture base bond. Chemical solvents are effective when they dissolve acrylic tooth-surface-enhancing bonds to denture base materials, and adhesive agents are effective in both acrylic and light-cured resin teeth.

Due to the emerging field of CAD/CAM and 3D-printing techniques for denture fabrication, different materials have been developed, for which little information still exists. From the investigated studies, it seems that depending on the teeth, DBR and bonding agents, bond strength can be similar to or lower than CCD [48]. Resins for heat-treated denture bases that were milled had better bond strengths than 3D-printed resins [45], while there is evidence that single-piece printed dentures can present higher bond strengths than CCD. Furthermore, photo-polymerized printed resins might be essentially weaker than pre-polymerized milled PMMA pucks [126]. Variations in printer resolution and curing wavelengths may affect the result, while the manufacturing of the pucks and the resulting porosities may be responsible for the differences in the milled DBR groups.

The strong bonding between teeth and the denture base is a benefit of single-piece CAD/CAM dentures. Other benefits are the quick production time, better materials properties in terms of roughness, porosity, flexural and impact strength, hardness, better retention, etc., and replacement is quick after fracture through patients’ digital records storage capability [136]. However, increased tooth wear has been recorded with that type of denture teeth, as have problems with the preservation of the occlusal vertical dimension [137]. The use of prefabricated artificial denture teeth with better physicochemical properties is instead made possible by technologies that allow the DBRs and teeth to be manufactured separately. As the increased amount of waste material and wear of milling burs constitute the most important drawbacks of CAD/CAM technology, improving 3D-printing technology is important, as it is a low-cost fabrication method. However, from the limited literature so far, it seems that the low strength and the poor optical and overall aesthetic properties, along with reduced retention [138], make this type of denture less attractive.

Due to controversial findings among the limited studies, further research should be performed to reveal if there is a clear trend in the prevalence of contemporary techniques compared with conventional ones. Furthermore, the diversity in applied methodologies to test the actual bond strength between denture teeth and denture bases, and the limited implementation of ISO guidelines, restricts the potential of a systematic evaluation of the findings from the included studies. Future research should be conducted including novel materials and processes to help clinicians eliminate the undesirable complication of teeth detachments from denture bases. At the same time, as ease of fabrication, possibility for multiple dentures printing with complex geometries and lower cost of 3D-printing dentures attract both technicians and clinicians more. Compared to conventional dentures fabrication, the optimization of bonding protocols of contemporary 3D-printed teeth are needed to ensure the durability of the bond.

## Figures and Tables

**Table 1:** Studies evaluating the bond between acrylic teeth and denture base materials.

Type of Chemical Treatment	Type of Mechanical Treatment	Type of Teeth	Type of DBRs	Reference
N/A	-220-grit sandpaper	-Acrylic teeth	-Heat-polymerized resin -Microwave-polymerized resin	Schneider et al., 2022 [10]
-MMA, 2 times, 30 s	-50 μm Al_2_O_3_, 2.5 bars, 10 s	-3D-printed acrylic teeth	-3D-printed DBR	Boonpitak et al., 2022 [39]
-Ethyl acetate -Bonding agent containing acetone and isopropyl alcohol	-Retentive holes, 2 mm × 0.5 mm	-Acrylic teeth	-Heat-polymerized resin	Al-Somaiday et al., 2022 [40]
-DCM -PMMA-based bonding agent	-Roughening with bur -250 μm Al_2_O_3_, 15 s, 4.8 bars, 10 mm	-Acrylic teeth -Composite teeth	-Heat-polymerized resin -CAD/CAM-milled	Helal et al., 2022 [41]
-MMA -3D-printed resin -Auto-polymerized acrylic resin	-400–1200-grit SiC paper	-3D-printed teeth -Prefabricated acrylic teeth	3D-printed denture resin Heat-polymerized resin	Cleto et al., 2022 [42]
Hot 3D tooth conditioning agent, 4 min, 40 °C + light-cured bonding agent	N/A	-3D-printed teeth -Prefabricated composite teeth -Milled teeth	-Heat-polymerized resin -Milled PMMA resin -3D-printed resin	Mohamed et al., 2022 [43]
PMMA bonding agent MMA	100 μm Al_2_O_3_, 1–2 bars	-3D-printed teeth -Acrylic teeth	-Heat-polymerized resin -3D-printed resin	Alharbi et al., 2021 [44]
PMMA bonding agent	50 μm Al_2_O_3_, 1–2 bars, 5 s	-Acrylic teeth	-Heat-polymerized resin -Milled PMMA resin -3D-printed resin	Kane et al., 2021 [45]
-PMMA-based bonding	Ν/A	-Acrylic teeth teeth -Milled teeth	-Heat-polymerized resin -Cold polymerized resin -Milled PMMA resin	Prpić et al., 2020 [46]
-Self-curing Bonding agent -Uncured 3D-printing resin	N/A	-Acrylic teeth teeth -3D-printed teeth	-Heat-polymerized resin -Milled PMMA resin -3D-printed resin	Choi et al., 2020 [47]
-4-META-based resin cement	-400–1200-grit SiC paper -50 μm Al_2_O_3_, 10 s, 2 bars, 10 mm	-Highly cross-linked -Composite with fillers	-Heat-polymerized -Pre-polymerized PMMA CAD/CAM resin	Han et al., 2020 [48]
-Ethyl acetate treatment	-Small T-shaped tunnel (1.6 mm) -Large T-shaped tunnel (2.1 mm)	-Composite teeth -Acrylic teeth	-Heat-polymerized resin -Thermoplastic resins	Tashiro et al., 2021 [49]
N/A	-50 μm Al_2_O_3_, 0.3 bars, 10 mm, 10 s -Diatoric cavity 2.3 mm × 2 mm	-Acrylic teeth	-Heat-polymerized resin	Sundaram et al., 2021 [50]
MMA DCM	-Groove 2 mm × 2 mm, straight, fissure bur -Groove 2 mm × 2 mm, inverted cone bur -Half round groove 2 mm, round bur	-Acrylic teeth	-Heat-polymerized resin	Kumar et al., 2021 [51]
-10% HF	-Grinding tungsten carbite, 40.000 rpm 2 mm grooves -50 μm Al_2_O_3_,10 s, 2.5 bar, 10 mm -Roughened with a flame-shaped diamond	-Acrylic teeth	-Heat-polymerized resin	Sayed et al., 2021 [52]
-MMA -DCM	-250 μm Al_2_O_3_, 4.8 bars, 5 s, 5 mm 5 mm	-Acrylic teeth	-Heat-polymerized resin	Viegas et al., 2021 [53]
-MMA, 30 s	-100% O_2_ and -100% N_2_ feeding gases (plasma), 30 min, 0.3 mbar, 60% power -250 µm Al_2_O_3_, 3 bars, 30 s, 5 mm	-Acrylic teeth	-Milled PMMA-based resin	Klaiber et al., 2021 [54]
-Polymer/monomer (3/1 *v*/*v*) -DCM, 45 s	-Grinding with sandpaper grit no −100 -T shaped groove 2 mm × 2 mm	-Acrylic teeth	-Heat-polymerized resin	Ravishankar et al., 2020 [55]
-MMA	N/A	-Acrylic teeth -Composite teeth -Nanocomposite teeth	-Heat-polymerized resin	Gharebagh et al., 2019 [56]
-MMA -DCM	-Al_2_O_3_ grinding stone	-Acrylic teeth	-Heat-polymerized resin	Sharma et al., 2019 [57]
-MMM -DCM, 30 s 1:1 *v*/*v* 30% TCM and MMM	N/A	-Acrylic teeth	-Heat-polymerized resin	Pathak et al., 2019 [58]
-MMA, 180 s	-Al_2_O_3_ 50 μm, 15 s, 5 mm -Vertical groove 2 mm × 2 mm	-Acrylic teeth	-Heat-polymerized resin -Thermoplastic resin	Prasad et al., 2018 [59]
-MMA	-Grinding with tungsten carbide bur, low speed	-Acrylic teeth	-Microwave- polymerized resin -Heat-polymerized resin	Aguiar et al., 2018 [60]
-DCM, 30 s	-Grinding with sandpaper grit no. −100 -L-shaped groove 2 mm × 2 mm wide	-Acrylic teeth	-Heat-polymerized resin	Phukela et al., 2018 [55]
-MMA, 180 s	-Diatoric cavity, 2 × 2.5 mm -Al_2_O_3_ 50 μm, 2 bars, 5 mm, 5 s	-Acrylic teeth	-Heat-polymerized resin	Hatami et al., 2017 [61]
N/A	-160 no. sandpaper -Retentive grooves 2 mm depth × 2 mm width	-Acrylic teeth	-Heat-polymerized resin	Mohamed et al., 2017 [62]
-1:1 MMA/DCM	-250 μm of Al_2_O_3_, 30 s at 4 p.s.i, -2 mm deep grooves	-Acrylic teeth	-Heat-polymerized resin	Mahadevan et al., 2015 [63]
N/A	-Al_2_O_3_ 50 μm, 15 s	-Acrylic teeth -Ceramic teeth	-Auto-polymerized resins -Heat-polymerized resin	Corsalini et al., 2014 [64]
-MMM -DCM	N/A	-Acrylic teeth	-Heat-polymerized resin	Jian et al., 2014 [65]
-MMA	-2.50 μm Al_2_O_3_, 8 bar,10 mm, 20 s	-Acrylic teeth	-Heat-polymerized resin	Chittaranjan et al., 2013 [66]
-Bonding agent	-Er:YAG laser	-Acrylic teeth	-Heat-polymerized resin -Light-polymerized resin	Akin et al., 2013 [67]
-MMA -Acetone -Chloroform -Cyanoacrylate -Ethyl acetate	N/A	-Acrylic teeth	-Heat-polymerized resins	Krishna et al., 2013 [68]
N/A	-Oxygen and argon plasma 2 min, 800 volt, 75 MA, 60 watt, 4 cm	-Acrylic teeth	-Heat-polymerized resin	Aljudy, 2013 [69]
-MMA	-Al_2_O_3_, 4.8 bars, 10 mm, 10 s -Diatoric cavity, 2 × 2.3 mm	Acrylic teeth	-Heat-polymerized resin	Meltoto et al., 2013 [70]
-MMA -ExpB -PB -VB	N/A	-Acrylic teeth	-Light-cured resin -Self-cured resin -Heat-cured resin	Palitsch et al., 2012 [71]
-MMA, 10 s	N/A	-Acrylic teeth	-Heat-polymerized resin	Kurt et al., 2012 [27]
-3/1 *v*/*v* Polymer/monomer	-Cingulum ledge lock (1 mm depth) -Diatoric hole (1.5 mm depth)	-Acrylic teeth	-Heat-polymerized resin	Phukela et al., 2011 [72]
-Acrylate bonding agent -MMA	-Grinding, tungsten carbide bur, 15,000 rpm -Diatoric undercut 1 × 2 mm^2^	-Acrylic teeth	-Heat-polymerized resin -Light-polymerized resin	Fletcher-Stark et al., 2011 [73]
-Methylacetate-based experimental bonding agent	-Grinding, tungsten carbide bur, 15,000 rpm -50 μm Al_2_O_3_, 5 bars, 5 mm, 5 s -Diatoric undercut 1.8 mm	-Acrylic teeth	-Auto-polymerized resin on heat-polymerized resin	Meng et al., 2010 [74]
-MMA	-#2000-grit grinding, 3000 rpm	-Acrylic teeth -Multilithic teeth	-Heat-polymerized resin	Mosharraf and Abed-Haghighi, 2009 [75]
-Bonding agent	320-, 400-, and 600-grit silicon carbide paper diamond-coated grinder, 18.000 rpm	-Acrylic teeth	-Heat-polymerized resin -Microwave-polymerized resin Light-cured resin	Marra et al., 2009 [76]
-MMA	N/A	-Acrylic teeth	-Heat-polymerized resin -Microwave-polymerized resin	Chaves et al., 2009 [77]
-MMA	-50 μm Al_2_O_3,_ 4.8 bars, 10 mm, 10 s -2.3 mm diameter -Al_2_O_3_ abrasive stone -Diatoric cavity (2 mm × 2.3 mm)	-Acrylic teeth	-Heat-polymerized resin	Bragaglia et al., 2009 [78]
N/A	-Grinding: tungsten carbide bur, 15,000 rpm -250 μm Al_2_O_3_ particles, 5 bars, 8 mm, 5 s.	-Acrylic teeth	-Heat-polymerized resin	Chung et al., 2008 [79]
-MMA, 60 and 180 s	N/A	-Acrylic teeth	-Auto-polymerized resin -Microwave-polymerized resin	Barbosa et al., 2008 [80]
-Methyl methacrylate-based bonding agent	-30 μm Al_2_O_3_ with silicon oxide, 2.8 bars, 10 mm, 20 s	-Acrylic teeth	-Heat-polymerized resin	Saavedra et al., 2007 [81]
N/A	-#1200 silicon carbide abrasive paper, laboratory sandblaster -50 μm Al_2_O_3_, 5 bars, 5 mm, 30 s -50 μm Al_2_O_3_, 5 bars, 5 mm. 30 s + adhesive primer	-Acrylic teeth	-Heat-polymerized resin	Nishigawa et al., 2006 [82]
MMA, 3 min composite bonding agent 37% phosphoric etchant + MMA MMA + composite bonding agent	-Grinding at low speed	-Acrylic teeth	-Auto-polymerized resin	Yanikoglou et al., 2002 [36]
-Ethylene glycol dimethacrylate monomer -MMA -Acrylic cements	-Medium grit rotary abrasion -Dovetail groove, 2 mm deep 3 2 mm wide	-Acrylic teeth	-Heat-polymerized resin	Cunningham et al., 1999 [83]
-MMA	-50 μm Al_2_O_3_, 20 s -Diatoric cavity 1.5 mm	-Acrylic teeth	-Heat-polymerized resin	Barpal et al., 1998 [84]
-10% hydrofluoric Acid -Silane coupling agent -Multipurpose bonding agent	-Laboratory sandblaster (6.2 bars, 50 μm Al_2_O_3_) -High-kinetic-energy sandblaster (6.2 bars, 50 μm Al_2_O_3_)	-Porcelain teeth	-Heat-polymerized resin	Merchack et al., 1995 [85]
-MMA, 180 s	-Grinding 120-grit sandpaper -Two grooves -Grinding and 1.5 mm diameter retention hole	-Acrylic teeth	-Heat-polymerized resin -Auto-polymerized resin	Valittu et al., 1995 [86]
N/A	-Milled ridge-lap into a cylinder 6 mm in diameter and 2 mm in length	-Acrylic teeth	Heat-polymerized resin Light-cured resin	Clancy et al., 1991 [87]
-MMA -Urethane dimethacrylate- based bonding agent	N/A	-Acrylic teeth -Multilithic teeth -IPN teeth	-Heat-polymerized resin -Auto-polymerized resin -Visible light-cured resin	Kawara et al., 1991 [88]
N/A	-Groove 1 mm depth	-Acrylic teeth	-Heat-polymerized resin	Clancy et al., 1989 [89]
-MMA	N/A	-Acrylic teeth	-Heat-polymerized resin	Spartley, 1987 [24]
N/A	-1 and 3 mm grinding	-Acrylic teeth	-Heat-polymerized resins	Caswell et al., 1986 [90]
-MMA, monomer–polymer solution 1/5 *v*/*v*	N/A	-Acrylic teeth	-Heat-polymerized resin	Morrow, 1978 [23]
-MMA (cold curing) -60% CHCI_3_ and 40% MMA -MA -50% GA and 50% acetone -60% DCM and 40% MMA containing 7.3 wt% PMMA -50% DCM and 50% MMA containing 10 %wt PMMA -50% DCM and 50% MMA containing 10 %wt PMMA -50% DCM and 50% MMA (cold curing)	N/A	-Acrylic teeth	-Cold-polymerized resin	Rupp et al., 1971 [29]

**Table 2:** List of studies reporting on the bond strength of 3D-printed or milled teeth with denture resin.

Author/Year	Materials	Method	Aging Process	Results	Conclusion
**Helal et al. 2022 [41]**	Denture base: -Heat-polymerized (Acrostone, Egypt) -CAD/CAM-milled (AvaDent PMMA, Scottsdale, AZ, USA) Denture teeth: -Composite (Eraylar-ostim, Ankara, Turkey) -Acrylic (Acrostone, Cairo, Egypt) Bonding agent: Ivobase CAD bond kit (Ivoclar Vivadent, Liechtenstein)	Static test: Shear bond strength test (SBS), universal testing machine, 0.5 mm/min Surface treatment: -No treatment (control) -Dichloromethane (DCM) -Roughening with bur -Alumina blasting; AB) n = 10 samples per group	N/A	Bond strength: Compared to other groups, DCM and AB resulted in increased SBS in acrylic teeth attached to heat-polymerized DBR In milled DBR with acrylic teeth, all surface treatments failed to significantly differ from one another SBS was significantly lower in composite teeth	When compared to the untreated group, AB and DCM use enhanced the SBS of acrylic teeth with heat-polymerized DBR, but none of the surface treatments demonstrated a discernible improvement with MIL DBR However, AB alone enhanced the SBS with heat-polymerized DBR, surface treatments resulted in decreased SBS in composite teeth combined with milled DBR
**Cleto et al. 2022 [42]**	Denture base: -3D-printed denture bases (Cosmos Denture, Yller Digital, Pelotas, Brazil) -Heat-polymerized resin (Lucitone 550, Dentsply Sirona, Charlotte, NC, USA) Denture teeth: - 3D-printed (Cosmos TEMP, Yller Digital, Brazil) -Prefabricated PMMA (Biotone, Dentsply Sirona, NC, USA) Bonding agent: -Methylmethacrylate monomer (MMA) (Duralay Reliance Dental Manufacturing, Alsip, IL, USA) -3D-printed resin (Cosmos TEMP, Pelotas, Brazil) -Auto-polymerized acrylic resin (Duralay, Reliance Dental Manufacturing, Il, USA)	Static test: Shear bond strength test (SBS), universal testing machine, 1 mm/min Groups: -Control (PMMA teeth + Heat-polymerized resin) -Heat-polymerized resin, PMMA teeth, MMA bonding agent Heat-polymerized resin, PMMA teeth, MMA bonding agent n = 10 samples per group	N/A	Bond strength: Highest values were recorded when 3D-printed DBR was combined with both PMMA and 3D-printed teeth bonded with the combination of MMA monomer and 3D-printed resin (comparable to heat-polymerized DBR with PMMA teeth) 3D-printed DBR combined with 3D-printed teeth that were bonded with the RD-printed resin bonding agent presented acceptable SBS	The type of teeth, as well as the bonding agent, influenced bond strength
**Mohamed et al. 2022 [43]**	Denture Base: -AvaDent -Lucitone digital print—original shade (Dentsply Sirona, NC, USA) -Acron, (GC, Tokyo, Japan) Denture teeth: -XCL1 -IPN 3D print tooth material (Dentsply Sirona, USA) -Endura Anterio HC5; Shofu Inc., Kyoto, Japan	Static test: Universal testing machine, 90° angulation of load to tooth, 1 mm/min cross-head speed n = 20 per group	Thermal cycling, 10,000 cycles 5 °C and 55 °C, immersion time 30 s, dwell time of 30 s	Before thermocycling: Statistically significant differences between MIL and CO, no statistically significant difference between PR and MIL or CO After thermocycling: MIL and PR groups presented significantly higher bond strength compared with CO Failure mode: MIL = 100% cohesive PR = CO 100% mixed failure mode	All methods presented adequate bond strength according to ISO 19736; milled and 3D-printed groups presented better results compared with CO even after thermal cycling.
**Boonpitak et al. 2022 [39]**	Denture Base: NextDent Denture 3D (3D Systems, Rock Hill, SC, USA) Denture teeth: NextDent C&B MFH (3D Systems, Rock Hill, SC, USA) Bonding agent: MMA (Palabond, Heraeus Kulzer, Hanau, Germany)	Static test: Universal testing machine, 90° angulation of load to tooth, 0.5 mm/min cross-head speed n = 10 per group	N/A	The shear bond strength was statistically significantly higher compared with the control group	When MMA was applied and then specimens were additionally heat cured, a higher bond strength was achieved
**Alharbi et al. 2021 [44]**	Test group: Denture base: E-Denture 3D+, EnvisionTEC, Dearborn, MI, USA Denture teeth: US denture teeth (E-Dent 400 C&B MFH, EnvisionTEC, MI, USA) Control group: Denture base: heat-cured PMMA (Major Base 20, Major Prodotti Dentari S.p.A., Torino, Moncalieri, Italy) Denture teeth: Trubyte Bioform IPN (Dentsply Sirona, USA) (compression-molding technique) Bonding agent: Ivobase CAD bond kit (Ivoclar Vivadent, Schaan, LIiechtenstein)	Static test after dynamic loading: Universal testing machine, ISO/TS 19736 50N load, 90° angulation of load to tooth, 1 mm/min cross-head speed n = 20 samples per group	Dynamic test: Chewing simulator at a 30°, horizontal speed 50 mm/s, 250.000 cycles, 50 N load	Bond strength: PR > CO Failure mode: CO: Cohesive PR: Mixed	Test group (3D-printed dentures) presented higher bond strength The bond strength was acceptable for both fabrication methods The bond strength of both groups was not considerably impacted by the chewing simulation
**Kane et al. 2021 [45]**	Denture Base: -Milled PMMA (Ivobase CAD, Ivoclar Vivadent, Liechtenstein -Milled PMMA (Polident, Renče-Vogrsko, Slovenia) -3D-printed resin (LP, Formlabs, Somerville, MA, USA -3D-printed resin (Lucitone Digital Print, Dentsply Sirona, NC, USA) -Conventional heat-polymerized PMMA (Lucitone 199, Dentsply Sirona, NC, USA) Denture teeth: -VITA Vitapan XL T44 (VITA Zahnfabrik, Bad Säckingen, Germany) -Denture tooth bonding agent (Ivobase CAD bond kit, Ivoclar Vivadent, LIiechtenstein)	Static test: universal testing machine, 90° angulation of load to tooth, 0.5 mm/min cross-head speed n = 9 samples per group	Specimens were aged in water for 600 h at 37 °C (2 y)	Bond strength: CO = MIL > PR Failure mode: Adhesive failures	Compared to 3D-printed bases, milled denture bases performed better but without differences compared to heat-polymerized ones The lower bond strength values of 3D-printed dentures may be attributed to the photopolymerization of resins compared to the pre-polymerized milled resins
**Prpić et al. 2020 [46]**	Denture base: -CAD/CAM (milled) DBR (Ivobase CAD, Ivoclar Vivadent, Liechtenstein) -Heat-polymerized DBR (Probase Hot, Ivoclar Vivadent, Liechtenstein) -Cold-polymerized DBR (Probase Cold, Ivoclar Vivadent, Liechtenstein) Denture teeth: -Nanohybrid composite teeth (Phonares II Typ, Ivoclar Vivadent, Liechtenstein) -Prefabricated acrylic teeth (SR Orthotyp S PE, Ivoclar Vivadent, Liechtenstein) -Cross-linked teeth (SR Orthotyp DCL, Ivoclar Vivadent, Liechtenstein) -CAD/CAM-milled (SR Vivadent CAD, Ivoclar Vivadent, Liechtenstein)	Shear bond strength test (SBS), universal testing machine, 0.5 mm/min n = 8 samples per group	Specimens were stored in ddH_2_O 48 h	Bond strength: The observed differences among groups were statistically significant 3.37 ± 2.14 MPa for CO with cold-polymerized resin 18.10 ± 2.68 MPa for CO with heat-polymerized resin Failure mode: CO with heat-polymerized resin, Mil: Cohesive/mixed CO with cold-polymerized resin: Adhesive	DBRs attached to various types of prefabricated teeth using CAD/CAM and heat-polymerized technology have comparable shear bond strength values Shear bond strength is highly dependent on the combination of teeth and DBR
**Choi et al. 2020 [47]**	Denture base: -3D-printed (Dima Print Denture Base, Kulzer, South Bend, IN, USA) -CAD-milled (Ivobase CAD, Ivoclar Vivadent, Liechtenstein) -Heat-polymerized (Vertex Rapid Simplified, Vertex-Dental, Soesterberg, The Netherlands) Denture teeth: -3D-printed resin teeth (Dima Print Denture Teeth, Kulzer, IN, USA) -Double-cross-linked PMMA (Ivoclar DCL, Ivoclar Vivadent, Liechtenstein) -PMMA with nanofillers (Mondial, Kulzer, IN, USA) -Unfilled PMMA (Ivoclar SPE, Ivoclar Vivadent, Liechtenstein)	Four-point flexure test using a universal testing machine Fracture toughness K_1C_ and flexure bond strength, ASTM C1421 n = 30 samples per group	Thermal cycling, 600 and 1200 cycles 5 °C and 55 °C, immersion time 30 s, dwell time of 30 s	Bond strength: CO > PR, Mil Failure mode: CO, PR: Adhesive failures MIL: Cohesive failures	The strongest bonds were created when teeth were bound to heat-polymerized DBRs, although they became much weaker with aging There was no significant effect of aging on the bond strength of teeth attached to CAD/CAM- and 3D-manufactured DBRs
**Han et al. 2020 [48]**	Denture base: -Heat-polymerizing (Vertex Rapid Simplified, Vertex-Dental, Netherlands) -Pre-polymerized CAD/CAM resin blocks (PMMA pink disc Block, Huge, China, and Vipi Block-Pink, Vipi Industria, Pirassununga, Brazil) Denture teeth: -Highly cross-linked (VITA MFT^®^, VITA Zahnfabrik, Germany) -Composite with fillers (1): Duracross Physio (Nissin Dental Products Inc., Kyoto, Japan) -Composite with fillers (2): Endura Posterio (Shofu Dental, Kyoto, Japan) Bonding agent: Resin cement (Super Bond C&B Sun medical, Moriyama-shi, Japan)	Shear bond strength test (SBS), universal testing machine, 0.5 mm/min n = 10 per group	Specimens were stored in ddH_2_O 24 h, ISO/TS 11405	Bond strength: There were no significant variations between groups except for composite teeth, which had the lowest SBS of all combinations. Failure mode: C = M Adhesive	Shear bond strengths developed between resin teeth and CAD/CAM denture base materials are equivalent to those achieved with traditional techniques

CO = conventional heat-polymerized DBR with prefabricated acrylic teeth, Mil = CAD/CAM-milled DBR, PR = 3D-printed DBR.

**Table 3:** Indicative 3D-printed resins and their compositions as stated in the corresponding safety data sheets.

Commercial Resins for DBR	Composition
-ucitone digital print-original shade (Dentsply Sirona, Charlotte, NC, USA)	Urethane Methacrylate 40–50% Organic Methacrylate Monomer 40–50% Organic Acrylate Monomer 1–5% Photoinitiator < 1.5%
Flexcera Base series (EnvisionTEC, MI, USA)	Diphenyl(2,4,6-trimethylbenzoyl)phosphine oxide 1–3 Methacrylated monomer 10–50 Methacrylated oligomer 30–80%
Formlabs denture base resins/teeth	Methacrylate monomer Urethane dimethacrylate Propylidynetrimethyl trimethacrylate Diphenyl(2,4,6- trimethylbenzoyl)phosphine oxide
NextDent Denture 3D+ (NextDent B.V. Soesterberg The Netherlands)	Monomer based on acrylic esters: Ethoxylated Bisphenol A > 60% *w*/*w* Methacrylic oligomer 15–25% Phosphine oxide < 2.5%
Optiprint laviva (dentona AG, Dortmund, Germany)	Methacrylate-based resin 3D-printing systems with 385 nm or 405 nm light sources for fabrication of dental bases: Aliphatic difunctional methacrylate < 40% Aliphatic urethane Acrylate < 10% 2-Propenoic acid, reaction products with pentaerythritol < 5% Cristobalitmehl < 20% 2,2’-ethylenedioxydiethyl dimethacrylate < 10% Siliziumdioxid < 6% diphenyl(2,4,6-trimethylbenzoyl)phosphine oxide < 2%
ASIGADentaBASE	7,7,9(or 7,9,9)-trimethyl-4,13-dioxo-3,14-dioxa-5,12-diazahexadecane-1,16-diyl bismethacrylate 10–25% Tetrahydrofurfuryl methacrylate 10–20% Diphenyl(2,4,6-trimethylbenzoyl) 10–20%
Denture (POWERRESINS, Istanbul, Turkey)	Esterification products of 4,4’-isopropylidenediphenol, ethoxylated and 2-methylprop-2-enoic acid 40–60% 7,7,9(or 7,9,9)-trimethyl-4,13-dioxo-3,14-dioxa-5,12-diazahexadecane-1,16-diyl bismethacrylate 10–20% Titanium dioxide 5–10% diphenyl(2,4,6-trimethylbenzoyl)phosphine oxide 1–5%
dima Print Denture Base (Kulzer, Hanau, Germany)	Esterification products of 4,4’-isopropylidenediphenol, ethoxylated and 2-methylprop-2-enoic acid 40–60% 7,7,9(or 7,9,9)-trimethyl-4,13-dioxo-3,14-dioxa-5,12-diazahexadecane-1,16-diyl bismethacrylate 30–50% Propylidynetrimethyl trimethacrylate 3–10% Diphenyl(2,4,6-trimethylbenzoyl)phosphine oxide < 3% Mequinol < 1%

## Data Availability

Not applicable.

## Appendix A

CCD	Conventional complete dentures
CAD/CAM	Computer-aided design and computer-aided manufacturing
SLA	Stereolithography
DLP	Digital light processing
DBR	Denture base resin
IPN	Interconnected penetrating network
SBS	Shear bond strength test
PMMA	Polymethyl-methacryalte
MMA	Methyl-methacrylate
GA	Glacial acetic acid
MA	Methacrylic acid
MMM	Monomethyl methacrylate monomer
4-META	4-Methacryloxyethyl trimellitate anhydride
ExpB	Mixture of Bis-GMA, TEGDMA, acetone and initiators
VB	Mixture of ethyl acetate, acetone, monofunctional and multifunctional methacrylate, acrylate, photoinitiator
PB	Mixture of methylmethacrylate, di-methacrylate, methacrylic acid
PEMA	polyethylmethacrylate
MRP	Microfillers polymerized into the matrix
UDMA	Urethane-dimethacrylate
DCM	Dichloromethane
TCM	Trichloromethane
